# Characteristics of adhesive bonding with enamel deproteinization

**DOI:** 10.1590/2177-6709.24.5.29.e1-8.onl

**Published:** 2019

**Authors:** Ghada Abdelaziz Mahmoud, Mohammed E. Grawish, Marwa Sameh Shamaa, Yasser Lotfy Abdelnaby

**Affiliations:** 1 Mansoura University, Faculty of Dentistry, Department of Orthodontics (Mansoura, Egypt).; 2 Mansoura University, Faculty of Dentistry, Department of Oral Biology (Mansoura, Egypt).

**Keywords:** Orthodontic brackets, Sodium hypochlorite, Electron microscopy.

## Abstract

**Objective:**

To evaluate the effect of using sodium hypochlorite (NaOCl) on the bond characteristics of orthodontic metal brackets bonded to enamel surface using three adhesive systems.

**Methods:**

One hundred twenty premolars were selected and randomly divided into two equal groups of 60 teeth/ each (Groups I and II). The teeth of Group I were left untreated while those of Group II were exposed to 5.25% NaOCl for 1 minute. The teeth in either group were randomly subdivided into three equal subgroups of 20 teeth/ each (A, B and C), according to the type of adhesive system used to bond the brackets. In Subgroup A, phosphoric acid + Transbond XT primer and adhesive were used. In subgroup B, Transbond Plus self-etching primer (SEP) + Transbond XT adhesive were utilized. In subgroup C, phosphoric acid + SmartBond LC adhesive were used. The shear bond strength (SBS) and the degree of adhesive penetration to enamel surface were assessed. Data analyses were performed using ANOVA, post-hoc (LSD), t and chi-square test.

**Results:**

Transbond XT had significantly higher SBS than SmartBond LC (*p*< 0.05). Phosphoric acid provided significantly higher SBS and degree of adhesive penetration than SEP (*p*< 0.05). NaOCl significantly increased SBS and degree of adhesive penetration in Subgroups B and C (*p*< 0.05).

**Conclusion:**

Adhesion quality of Transbond XT adhesive is better than SmartBond LC. Phosphoric acid is more effective than SEP. NaOCl enhances the bond characteristics.

## RESUMO

**Objetivo:**

avaliar os efeitos do uso de hipoclorito de sódio (NaOCl) sobre as características de colagem de braquetes ortodônticos metálicos na superfície do esmalte, utilizando três sistemas adesivos.

**Métodos:**

cento e vinte pré-molares foram selecionados e divididos aleatoriamente em dois grupos iguais, cada um com 60 dentes (Grupos I e II). Os dentes do Grupo I não foram tratados, enquanto os do Grupo II foram expostos ao NaOCl a 5,25% por 1 minuto. Os dentes de ambos os grupos foram subdivididos, aleatoriamente, em três subgrupos iguais, cada um com 20 dentes (A, B e C), de acordo com o tipo de sistema adesivo utilizado para colar os braquetes. No Subgrupo A, foi utilizada a combinação ácido fosfórico + Transbond XT (*primer* e adesivo). No Subgrupo B, foi utilizada a combinação Transbond Plus autocondicionante (SEP) + Transbond XT (adesivo). No Subgrupo C, foi utilizada a combinação ácido fosfórico + SmartBond LC (adesivo). Foram avaliados a resistência ao cisalhamento e o grau de penetração do adesivo na superfície do esmalte. A análise dos dados foi realizada utilizando-se os testes ANOVA, *post-hoc* (LSD), *t* e qui-quadrado.

**Resultados:**

o Transbond XT teve uma força de adesão significativamente maior do que o SmartBond LC (*p*< 0,05). O ácido fosfórico propiciou força de adesão e grau de penetração do adesivo significativamente maiores do que o SEP (*p*< 0,05). O NaOCl aumentou significativamente a força de adesão e o grau de penetração do adesivo nos Subgrupos B e C (*p*< 0,05).

**Conclusão:**

a qualidade da adesão do adesivo Transbond XT é melhor que a do SmartBond LC. O ácido fosfórico é mais eficiente do que o SEP. O NaOCl melhora as características da colagem.

## INTRODUCTION

The traditional technique of orthodontic bracket bonding depends on acid etching of enamel surface, application of primer and utilization of adhesive resin. The success of such technique is influenced by the bond strength between brackets and enamel surface. The bond strength should be enough to withstand the orthodontic and masticatory forces.^1^ ^,^ ^2^

Several mechanical and chemical elements have been used to improve enamel retentive properties. Air abrasion and laser are examples of mechanical methods, but the obtained results were not satisfactory.^3^ ^,^ ^4^ Different acids with different concentrations have been utilized for enamel etching. However, the use of 32% to 40% phosphoric acid conditioner is still the best option to achieve predictable bonding to enamel.^5^ The goal of introducing self-etching primer (SEP) was to combine both conditioning and priming agents into one component, aiming to simplify the bonding procedures.^6^ However, SEP provides lower bond strength than the conventional acid etching technique.^7^ ^-^ ^9^

A different chemical method to enhance the etching pattern was described by Espinosa et al,^10^ who reported that wetting enamel surface before acid etching with 5.25% sodium hypochlorite (NaOCl) for 1 minute increased etching pattern quality, because NaOCl eliminates organic matter from enamel surface and acts as a deproteinizing agent. However, there were arguments about its effect on the bond strength.^11^ ^-^ ^13^

Composite resin is the most popular adhesive used for bracket bonding. However, several innovations have been introduced, such as cyanoacrylate adhesive. It doesn’t require the use of a primer or a curing light during bonding. This adhesive is activated when it comes in contact with a wet tooth surface. However, it provided lesser bond strength than other conventional adhesives.^14^ ^,^ ^15^ SmartBond light-cured cyanoacrylate adhesives can bond both dry and moist enamel, and it provides clinically acceptable bond strength especially in saliva contaminated situations.^16^

Since the bond strength of the adhesives is of great importance for orthodontic bracket survival, the present study was conducted to evaluate the effect of enamel deproteinization using 5.25% NaOCL on bond characteristics of metallic orthodontic brackets bonded with light-cured cyanoacrylate or composite resin adhesives after enamel conditioning with either 37% phosphoric acid or SEP.

## MATERIAL AND METHODS

One hundred twenty premolars extracted for orthodontic purposes were collected according to the following criteria: intact buccal enamel surface with no cracks, not subjected to pretreatment with chemical agents and free from any developmental defects or hypomineralized lesion or caries. The teeth were cleaned from any remnants and stored in 1% (w/v) thymol solution at room temperature. Metal brackets (Ormco, Glendora, CA, USA) were bonded to premolars’ buccal surfaces. Before bracket bonding, the premolars were randomly divided into two equal groups of 60 teeth each (Groups I and II). The buccal surfaces of the premolars in Group I were left untreated, while those of Group II were exposed to 5.25% NaOCl deproteinizing agent for 1 minute, utilizing a microbrush, then rinsing and drying for 10 seconds. The teeth in either group were further subdivided into three equal subgroups of 20 teeth each (Subgroups A, B and C) according to the adhesive system used (Table 1). Ten teeth from each subgroup were used to test the SBS. The other ten teeth were investigated with SEM to test the average length and total area percentage of adhesive penetration into enamel surface.

**Table 1:** Composition and manufacturer of the adhesive systems used in the present study.

Product	Composition	Manufacturer	Lot number
Ivoclar etchant gel	37% phosphoric acid as etchant.	IvoclarVivadent, Schaan, Liechtenstein	V06458
Transbond XT primer	Triethylene glycol dimethacrylate, bisphenol A diglycidyl ether dimethacrylate	3M Unitek, Monrovia, Calif	N715508
Transbond XT adhesive	Silane-treated quartz, bisphenol A diglycidyl ether dimethaacrylate, bisphenol A bis(2-hydroxyethyl ether )dimethacrylate, dichlorodimethylsilane reaction product with silica	3M Unitek, Monrovia, Calif	N720584
Transbond Plus SEP	Polyethylene glycol dimethacrylate, citric acid dimethacrylate oligomer, silane-treated quartz, glass reacted with hydrolyzed silane, silane-treated silica and bisphenol A diglycidyl ether dimethacrylate	3M Unitek, Monrovia, Calif	606648B
SmartBond LC	Poly methylmethacrylate, Ethyl cyanocrylate, silica. amorphous treated, hydroquinone	Gestenco, Gothenburg, Sweden	BAA92263

The sample size was determined using MedCalc Statistical Software version 14.8.1. A sample size of 9 teeth was required to detect a difference of 2.5 in the shear stress at Maximum Shear load between the two groups with NaOCl (SD =1.9) and without NaOCl application (SD = 1.6), assuming a significance level of 0.05 and 80% study power. The sample size was further adjusted to account for about 10% expected dropout rate. So, the final sample size was 10 teeth in each subgroup.

### Bonding procedures

In Subgroup A, the enamel surface was cleaned using a non-fluoridated pumice and rubber polishing cup, rinsed and dried for 10 seconds. Then, the enamel surface was conditioned using 37% N-Etch phosphoric acid gel (Ivoclar, Vivadent, Schaan, Liechtenstein) for 30 seconds, rinsed with water for 10 seconds and then dried with compressed oil-free air. The etched enamel was coated with thin layer of Transbond XT primer (3M Unitek, California, USA) using a microbrush. Transbond XT adhesive paste (3M Unitek) was applied to the bracket base and pressed firmly in the correct position on buccal surfaces of the teeth. Removing excess adhesive flash around the bracket base was done using sharp scaler and the adhesive was light-cured using Elipar S10 LED light-curing (3M ESPE, St. Paul, USA) for 20 seconds. In Subgroup B, the same steps were followed as in Subgroup A, except Transbond Plus SEP (3M Unitek) was used instead of phosphoric acid and Transbond XT primer. It was rubbed on the enamel buccal surface for 3 seconds and evaporated with gentle air. In Subgroup C, enamel surfaces were etched as in Subgroup A, except that, after etching, the surface was kept wet. SmartBond LC adhesive (Gestenco, Gothenburg, Sweden) was applied to the bracket base. Then the bracket was placed in its correct position and light-cured. The applications of all adhesives were done according to the manufacturer’s instructions. All samples were kept in an incubator with 37 °C temperature for 24 hours, then thermocycled between 5°C and 55°C for 500 cycles.^17^

### Shear bond strength testing

A customized self-cure acrylic block was used to mount each tooth utilizing polypropylene pipe. The teeth were completely embedded in the acrylic resin, leaving the buccal surface exposed. The acrylic blocks were fixed to the universal testing machine base (Model LRX-plus, Lloyd Instruments Ltd, Fareham, UK). The shear force was applied via knife-edge stainless steel blade attached to the upper compartment of the machine. The force was applied to the bracket-tooth interface in an occluso-gingival direction at a crosshead speed of 0.5 mm/minute until the bracket was detached. The force required for each bracket to dislodge was recorded in newtons (N) and SBS was calculated in megapascals (MPa) through dividing the force by bracket base surface area.

### Assessment of adhesive remnant index

All teeth were examined under stereomicroscope (SZ-PT, Olympus, Japan) after debonding, to assess the adhesive remnant index (ARI) on enamel surface, at a magnification of 10x. The scores of ARI ranged from 0 to 3, with 0 indicating no adhesive left on the enamel; 1, less than half of the adhesive left; 2, more than half of the adhesive left; and 3, all of the adhesive remained on the enamel surface.^18^

### Scanning electron microscope investigation

The teeth with the brackets in position were split vertically in bucco-palatal direction with a slow-speed water-cooled diamond saw (Isomet, 4000 micro saw, Buehler, USA), to obtain a specimen of 2 mm thickness, then the medial surfaces of the specimens were cleaned with distilled water in ultrasonic agitation for 30 minutes. The specimens were immersed in 37% HCL acid solution for 2 seconds, rinsed in distilled water and air dried, to remove the smear layer. The specimens were mounted on aluminum stubs and sputter coated with gold, and investigated using SEM (JSM-6510 LV, JEOL, Tokyo, Japan) operated at an accelerating voltage of 30 kV, at magnification from 40 to 6000 times. To standardize the microscopic observations, ten samples of each subgroup were scanned from occlusal to the cemento-enamel areas and therefore the relevant area of interest could be obtained. The resin tag analysis was done on SEM micrographs at 1200x magnification, by measuring resin tags average length and surface area % penetration into the enamel (in micrometers), utilizing Video Test Morphology^®^ software (Russian Federation) with a specific built-in routine for area, % area measurement and object counting.

### Statistical analysis

Data analyses were performed using SPSS version 16. The normality of distribution was evaluated using Shapiro-Wilk statistical test, and the homogeneity of variance was tested using Levene’s test. ANOVA test and LSD *post-hoc* tests were utilized to determine the significant differences among subgroups in each group. Also, t-test was used for two group comparisons (with and without application of NaOCl). The significant difference in the ARI scores was assessed via Chi-square test. The significance level was predetermined at *p*< 0.05.

## RESULTS

### Shear bond strength

The means, standard deviations of SBS values and results of the LSD *post-hoc* for the tested groups with and without NaOCl application are shown in Table 2. ANOVA test revealed an overall significant difference in SBS between the three adhesive systems in each group (*p*< 0.05). Transbond XT primer + adhesive provided the highest SBS (*p*< 0.05). Also, Transbond Plus SEP + Transbond XT adhesive had significantly higher bond strength than SmartBond LC adhesive. The utilization of NaOCl led to an increase in the SBS for all subgroups. Though, this increase was significant only with Transbond Plus SEP + Transbond XT and SmartBond LC adhesives (*p*< 0.05).

**Table 2:** Means, standard deviations of the SBS (MPa) and results of the LSD post-hoc and t tests for all studied subgroups.

Adhesive systems	Mean SBS and SD		t test	
	Without NaOCl	With NaOCl	t	P*
Transbond XT primer+adhesive	13.48 ± 2.79^bc^	14.54 ± 2.76^bc^	0.858	0.402
Transbond XT Plus SEP+ Transbond XT adhesive	6.06 ± 1.25^ac^	9.19 ± 2.47^ac^	3.581	0.002
SmartBond LC adhesive	4.27 ± 1.38^ab^	6.35 ± 2.13^ab^	2.588	0.019

In each column, means with the same superscript letters are not significantly different according to LSD post-hoc test. Significance p<0.05.

### Adhesive remnant index

ARI scores of the tested groups with and without NaOCl application are shown in Table 3. In general, the failure was mostly cohesive. However, failure at adhesive-bracket interface was mainly found in Transbond XT adhesive with application of NaOCl. Failure at adhesive-enamel interface was mainly found in SmartBond LC without application of NaOCl. The chi-square test revealed insignificant difference in ARI scores (*p*> 0.05).

**Table 3:** The ARI scores of the studied adhesive systems and the results of Chi-square test.

Adhesive systems	ARI scores									
	Without NaOCl				With NaOCl				For each adhesive	
	0	1	2	3	0	1	2	3	Chi-square	P*
Transbond XT primer+ Transbond XT adhesive	0	2	5	3	0	1	4	5	0.944	0.714
Transbond XT Plus SEP+ Transbond XT adhesive	2	4	3	1	1	3	4	2	0.952	1
SmartBond LC adhesive	6	2	1	1	1	4	3	2	5.571	0.178
	Chi-square = 12.267 P = 0.051			Chi-square = 4.932 P = 0.622						

0, no adhesive left on the enamel; 1, less than 50% of the adhesive left on the enamel; 2, more than 50% of the adhesive left on the enamel; 3 all adhesive left on the enamel.

### Scanning electron microscope evaluation

**Figure 1:**
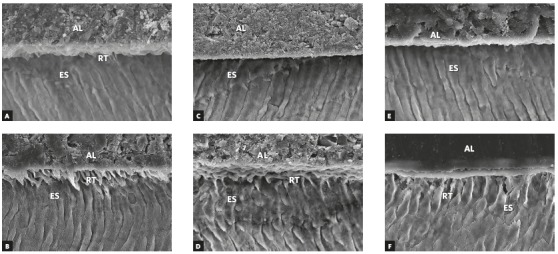
The adhesive-enamel interface at 1200x magnification using SEM. The brackets were bonded to enamel utilizing Transbond XT primer and adhesive (A), Transbond Plus SEP and Transbond XT adhesive (C), SmartBond LC adhesive (E). Deproteinization with 5.25% NaOCl was done and the brackets were bonded utilizing Transbond XT primer and adhesive (B), Transbond Plus SEP and Transbond XT adhesive (D) and SmartBond LC adhesive (F). Note the numerous long and thick resin tags that had penetrated into the enamel surface treated with NaOCl and bonded with Transbond XT adhesive. Meanwhile, few, short and thin resin tags had penetrated the untreated enamel surface bonded with SmartBond LC adhesive. In addition, moderate amount of penetration occurred in the specimens treated with NaOCl and bonded with Transbond XT plus SEP (AL = adhesive layer; ES = enamel surface; RT = resin tag).

According to ANOVA, there were significant differences in average length and total area % of resin tags penetration to enamel surface. Transbond XT primer + adhesive provided higher values in comparison to Transbond Plus SEP + Transbond XT and SmartBond LC adhesives in each group (*p*< 0.05). However, there was no significant difference between Transbond Plus SEP + Transbond XT and SmartBond LC adhesives in each group (*p*> 0.05). Regarding NaOCl application, SEM micrographs of the enamel-adhesive interface showed numerous, longer and thicker resin tags that had penetrated into enamel surface than those of Group I (without application of NaOCl). Also, the results of LSD test illustrated that with application of NaOCl, there was insignificant increase in average length, total area % of resin tags penetration to enamel surface bonded with Transbond XT primer + adhesive (*p*> 0.05). However, Subgroups B and C showed significant increase (*p*< 0.05) (Fig 1, Tables 4 and 5).

**Table 4:** Means, standard deviations of the average length (µm) of the resin tags penetration to enamel surface and results of the LSD post-hoc and t tests for all studied subgroups.

Adhesive systems	Mean Average length and SD			t test
	Without NaOCl	With NaOCl	t	P*
Transbond XT primer+ Transbond XT adhesive	11.09 ± 2.80^bc^	12.97 ± 1.87^bc^	1.765	0.095
Transbond XT Plus SEP+ Transbond XT adhesive	6.30 ± 1.85^a^	9.52 ± 2.29^a^	3.460	0.003
SmartBond LC adhesive	4.54 ± 1.01^a^	9.16 ± 1.09^a^	9.830	<0.001

In each column means with the same superscript letters are not significantly different according to LSD post-hoc test. Significance: P<0.05.

**Table 5:** Means, standard deviations of the Total area (%) of the resin tags penetration to enamel surface and results of the LSD post-hoc and t tests for all studied subgroups.

Adhesive systems	Mean total area % and SD	t test		
	Without NaOCl	With NaOCl	t	P*
Transbond XT primer+ Transbond XT adhesive	10.94 ± 2.09^bc^	12.52 ± 1.67^bc^	1.870	0.078
Transbond XT Plus SEP+ Transbond XT adhesive	6.40 ± 2.54^a^	9.92 ± 1.79^a^	3.591	0.002
SmartBond LC adhesive	4.79 ± 1.03^a^	9.20 ± 1.62^a^	7.269	<0.001

In each column means with the same superscript letters are not significantly different according to LSD post-hoc test. Significance: P<0.05.

## DISCUSSION

In the present study, the significant higher SBS of Transbond XT adhesive in comparison to SmartBond LC adhesive could be attributed to the difference in composition and strength between the two adhesives. Shinchi et al^19^ reported that the bond strength between adhesive and enamel surface was affected by resin strength and its ability to penetrate the crystalline enamel surface. In addition, according to SEM results, the penetration of Transbond XT adhesive was more pronounced than SmartBond LC and this could be due to different viscosity of the adhesives, which could affect the bond strength. This finding was in agreement with those of Al-Munajed et al,^20^ who found that the SBS values for cyanoacrylate were significantly lower than for composite resin. On the other hand, the present results were not in harmony with those of Örtendahl and Örtengren^14^ and Bishara et al.^21^ This disharmony could be attributed to different methodology, since the later studies didn’t perform thermocycling. Bishara et al^15^ illustrated that cyanoacrylate SBS decreased by 80% after thermocycling between 5°C and 55°C. In addition, Cacciafesta et al^16^ found that in wet condition, SmartBond LC showed greater bond strength (particularly under saliva contamination), in comparison to Transbond XT. The inherent hydrophilic property of cyanoacrylate adhesive would be responsible for this effect. Therefore, it may be advantageous to use SmartBond LC in certain clinical situations in which is difficult to achieve moisture control.

The use of traditional etching in the present study provided higher SBS than Transbond Plus SEP. This could be attributed to lower pH of phosphoric acid than SEP, which provides a higher ability to turn the surface from a low energy hydrophobic to a high energy hydrophilic, facilitating penetration into the enamel surface.^22^ The SEM results of this study revealed that the average length and surface area % of the resin tags were greater with utilization of conventional etch (11.09 ± 2.80µm) than with SEP (6.30 ± 1.85µm) and this result confirms the result of the SBS. The present finding was in harmony with those of Ireland et al^23^ and Aljubouri et al.^24^ On the contrary, Miyazaki et al^25^ and Reis et al^26^ found similar SBS with utilization of either SEP or total etch adhesives.

Regarding NaOCl effect, 5.25% NaOCl application increased SBS for all adhesive systems. This increase was significant in Subgroup B (Transbond Plus SEP + Transbond adhesive) and Subgroup C (phosphoric acid + SmartBond LC). This finding could be attributed to the presence of the acquired pellicle that covers the tooth enamel surface, which acts as a barrier, preventing composite from adhering to enamel surface and therefore compromising the bond strength between tooth surface and orthodontic brackets.^27^ Accordingly using NaOCl before etching could improve the bond strength through removal of such element. In addition, the enamel etching pattern could be considered another important factor. Three different etching patterns were identified. In the type 1 etching pattern, phosphoric acid dissolves the head of the prism, with the peripheral material or interprismatic substance remaining intact. In type 2, the acid dilutes the peripheral zone of the prisms, leaving the prism head relatively intact. In type 3, the surface change has no specific features, but generally displays some superficial dissolution that does not alter the deeper strata where the enamel prisms are located. The most retentive etching patterns were types 1 and 2, because the porous surface offered retentive areas of greater size and depth. The type 3 etching pattern did not present a defined and deep morphology and lacked the micromechanical retention, offered by the previous two. Therefore, it is desirable to obtain types 1 and 2 etching patterns.^28^ Espinosa et al^12^ concluded that more type 1 and 2 etching patterns were found when enamel surface was deproteinize with NaOCl, while type 3 etching pattern was found without deproteinization with NaOCl. Furthermore, according to SEM results, the enamel surface that was treated with NaOCl and then with phosphoric acid showed higher penetration and surface area than non-conditioned enamel. The results of the present study regarding the effect of NaOCl on enhancement of the SBS was in line with those of other authors.^10^ ^-^ ^12^ On the other hand, Ahuja et al^13^ found that deproteinization using 5.25% NaOCl had no influence on type 1 and 2 etching patterns.

The SBS value of SmartBond LC adhesive after deproteiniztion with NaOCl (6.35 ± 2.13) was significantly higher and lie within the accepted clinical range recommended by Reynolds^2^ (6-8 MPa).Also, there was pronounced enhancement in SBS of Transbond Plus SEP (9.19 ± 2.47). Thus, from the clinical point of view it’s advisable to deproteinize the enamel surface with 5.25% NaOCl for 1 min before the utilization of either SEP or SmartBond LC, to enhance the bond strength, in spite of increasing the steps of the bonding procedures. However, care should be taken in extrapolating such results to those that might be obtained in the oral environment. 

The ARI scores revealed that no significant differences were found between all adhesive systems. For all subgroups, debonded brackets showed failure at adhesive-enamel interface as well as at adhesive-bracket interface. In general, bracket failure at adhesive-bracket interface is favorable as it reveals good adhesion to the enamel surface. In contrast, to remove the residual adhesive, considerable chair time is needed. Also, during cleaning process, the possibility of damaging enamel surface is increased.^29^ On the other hand, when brackets failure occurs at adhesive-enamel interface, less residual adhesive remains on enamel surface, bracket failure probably take place during treatment, with disturbing chair time and leading to lengthening of orthodontic treatment duration.^30^

## CONCLUSION

» Transbond XT adhesive had higher SBS than SmartBond LC adhesive.
» The utilization of the conventional etching (phosphoric acid and Transbond XT primer) provided higher SBS and adhesive penetration (average length and total area %) into enamel surface than Transbond Plus SEP.
» Enamel deprotenization with 5.25% NaOCl for 1 minute before enamel etching increased the SBS and adhesive penetration to enamel surface.
